# MRI features that predict progression of residual disease after ablation of extra-abdominal desmoid fibromatosis

**DOI:** 10.1007/s00330-024-11319-w

**Published:** 2025-01-21

**Authors:** Daniel M. Düx, Yosef Chodakiewitz, Rachelle Bitton, Sharmila Sewell, Vipul R. Sheth, Pejman Ghanouni, Ryan L. Brunsing

**Affiliations:** 1 Department of Radiology, Hannover Medical School (MHH), Carl-Neuberg-Straße 1, 30625 Hannover, Germany; 2 Department of Radiology, Stanford School of Medicine, Stanford, CA 94305 USA

**Keywords:** Fibromatosis (aggressive), Ablation techniques, Neoplasms (connective and soft tissue), Musculoskeletal diseases, Magnetic resonance imaging

## Abstract

**Objective:**

To identify MRI features of desmoid tumors (DTs) that predict the growth of residual disease following ablation.

**Methods:**

Patients who underwent MRI-guided ablation for DTs between February 2013 and April 2021 were included in this single-center IRB-approved retrospective study. MRI scans assessed three suspicious tissue features: intermediate T2 signal [+iT2], nodular appearance [+NOD], and contrast enhancement [+ENH]. Percent-monthly change in diameter (PMCD) of suspicious foci determined growth (PMCD > 1%), unchanged (PMCD between −1% and +1%), or regression (PMCD < −1%). Statistical tests compared mean PMCD between groups and evaluated sensitivity and specificity.

**Results:**

Thirty-three patients (32 years ± 13.3; 22 females) with 34 DTs underwent 47 MRI-guided ablations, with a median follow-up of 269 days (IQR 147). Of 93 suspicious foci, 62 (67%) grew (PMCD: +5.6% IQR: 5.8), 13 (14%) remained unchanged (PMCD: −0.1% IQR: 0.6), and 18 (19%) regressed (PMCD: −3.9% IQR: 4.2). Features [+iT2], [+ENH], and [+NOD] were associated with PMCDs of +5.2% IQR: 6.0, +3.4% IQR: 6.0, and +3.4% IQR: 6.5, respectively, compared to −1.5% IQR: 4.7 (*p* < 0.0001), −0.5% IQR: 0.8 (*p* = 0.003), and +0.4% IQR: 7.5 (*p* = 0.0056) for their respective negative counterparts. Sensitivity, specificity, and accuracy for distinguishing growth were [+iT2]: 0.95, 0.71, 0.87, [+ENH]: 1.00, 0.32, 0.77, and [+NOD]: 0.84, 0.42, 0.70. Combining [+iT2 + NOD + ENH] yielded PMCD +5.9% IQR: 6.2 and the best performance for distinguishing growth (sensitivity 0.81, specificity 0.94, accuracy 0.85).

**Discussion:**

MRI features reliably predict the growth of residual or recurrent DTs post-ablation, with [+iT2] being the most accurate. Adding nodular enhancement to [+iT2] improved specificity without sacrificing accuracy.

**Key Points:**

***Question*** *Post-ablation imaging of desmoids is challenging due to tumor heterogeneity and treatment-related inflammation. This study evaluates MRI features for assessing future tumor growth.*

***Findings*** *Foci of intermediate T2 signal post-ablation predicted desmoid growth with high sensitivity (0.95), while T2 signal, nodularity, and enhancement combined offer high specificity (0.94).*

***Clinical relevance*** *Intermediate T2 signal predicts desmoid tumor growth post-ablation with high sensitivity and accuracy but moderate specificity. Combining nodularity and enhancement improves specificity and predictive value, helping clinicians in managing desmoid tumor patients post-ablation.*

## Introduction

Desmoid tumors (DTs) are rare benign fibroblastic soft tissue tumors predominantly affecting young patients [1–3]. Their locally infiltrative nature often leads to pain and functional impairment, contributing to significant morbidity [4]. Traditional treatments like surgery, radiation, and chemotherapy can be invasive and carry risks of complications or secondary malignancies [4]. Focal ablation, including techniques like cryoablation and high-intensity focused ultrasound (HIFU) guided by MRI, offers a less invasive alternative with potentially fewer side effects [5–8]. Recognizing its benefits, focal ablation has been incorporated into the 2021 National Comprehensive Cancer Network (NCCN) guidelines as a primary treatment option for DTs [9].

However, evaluating treatment response and detecting residual or recurrent disease post-ablation remains challenging. Common assessment criteria (e.g., response evaluation criteria in solid tumors (RECIST), WHO criteria, and/or volumetrics) evaluating tumor size may have suboptimal sensitivity in predicting response in DTs [10–13]. RECIST and modified response evaluation criteria in solid tumors (mRECIST) criteria are often used to assess DTs [10, 11], however both suffer from low intra- and interobserver agreement, even with systemic therapy [10]. This raises questions about the mRECIST utility of DTs in the post-ablation setting, where ablation cavity contraction, inflammation, and growth along ablation margins can impact perceived lesion size. Changes in lesion size alone may not reflect treatment efficacy due to post-ablation tissue alterations such as inflammation or cavity formation [12]. This underscores the importance of employing advanced imaging modalities, specifically MRI, which can capture subtle changes in DT characteristics beyond mere size metrics.

Various MRI features, such as modified Choi criteria, T2 signal intensity, and enhancement patterns, have shown promise in assessing treatment response and predicting tumor behavior in DTs [10–12, 14–16]. Nonetheless, the literature exhibits variability, particularly concerning the predictive value of T2 signal intensity [13, 17, 18]. While some studies found T2 signal hyperintensity as predictive of tumor growth [14–16], others did not [13, 17]. However, these investigations focused on the entire tumor, rather than specific foci within the tumor, which may exhibit variable behavior post-ablation, and used different approaches to T2 signal intensity classification. Moreover, post-ablation inflammation can confound imaging interpretation, mimicking viable tumor tissue [12].

This study aims to investigate whether combining key MRI features—T2 signal intensity, enhancement characteristics, and nodularity—can collectively enhance the prediction of extra-abdominal DT growth following MRI-guided focal ablation. By elucidating the relationship between these imaging markers and tumor behavior post-treatment, this research seeks to refine the clinical management and surveillance strategies for patients with DTs undergoing focal ablation.

## Material and methods

### Patient cohort

This IRB-approved retrospective single-center study identified patients with biopsy-proven extra-abdominal desmoid tumors (DTs) treated using MR-guided focused ultrasound (MRgFUS) ablation or MRI-guided cryoablation between February 2013 and April 2021. Demographic data were retrieved from electronic medical records. The study included patients meeting the following criteria:

Inclusion:

- DTs treated with MRI-guided focal ablation
- At least two follow-up MRIs within 12 ± 2 months post-treatment meeting specified technical requirements
- Measurable lesions within the treatment area on follow-up MRIs

Exclusion:

- Patients lacking post-ablation MRI scans

### Hardware and technical settings

MRgFUS was performed with the ExAblate 2100 System (INSIGHTEC) and MRI-guided cryoablations with the VISUAL ICE™ System (Boston Scientific). All procedures were performed on 3-Tesla MRI scanners (750w or Architect, GE Healthcare). Pre- and post-treatment MRI had to include T2-weighted imaging ± fat suppression (repetition time (TR) 5 s, echo time (TE) 60 ms, field of view (FOV) 22 cm, slice thickness 5 mm, matrix 320 × 192), T1 weighted fast spin echo imaging without fat suppression (TR 5.7 ms, TE 1.88 ms, FOV 30 cm, slice thickness 4 mm, matrix 176 × 224), and pre- and post-contrast T1 weighted 3D spoiled gradient recalled echo (SPGR) imaging with Dixon-based fat suppression (TR ~ 4.2 ms, TE ~ 1.7 ms, FOV ~ 38 cm, slice thickness 1.5 mm, matrix 320 × 256).

### Image analysis

**Fig. 1:**
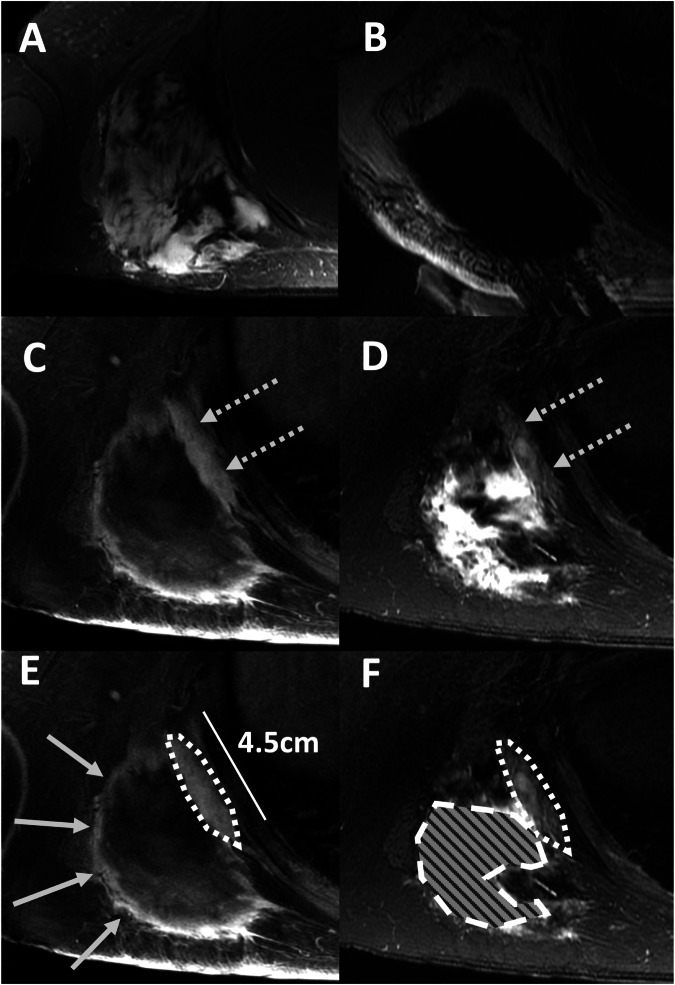
MR-guided cryoablation and post-ablation MRI features in follow-up MRI. **A** Desmoid tumor in the right shoulder of a 30-year-old female (14 × 9 × 12 cm), causing significant pain and limiting motion of the scapula (axial T1 weighted post-contrast image). **B** Cryoablation: Treatment of the tumor, which included a pullback and second round of treatment (Axial PD weighted images). **C**, **D** Residual disease (dashed arrows) along the chest wall measuring 4.5 cm in follow-up MRI 263 days following cryoablation (axial T1 weighted SPGR post-contrast (**C**) and axial T2 weighted SSFSE with fat suppression images (**D**)). **E**, **F** Key post-ablation imaging features used in the paper, with contours overlaid on images **C** and **D**. The dotted outline shows desmoid tissue that has intermediate T2 signal [+iT2] and enhances [+ENH], but appears linear and not nodular [−NOD]. The dashed outline filled with a pattern shows an example of an area of marked (fluid-like) T2 hyperintense areas that was attributed to post-ablation changes and not considered +iT2 signal. The arrows point to a region that only exhibits +ENH. Examples of nodularity [+NOD] are shown in later figures

Post-ablation MRIs within 12 ± 2 months post-treatment were analyzed. Treatment initiation was designated as *t* = 0, with subsequent MRIs at *t* = 1, 2, 3, etc. Baseline lesions could manifest on the immediate post-ablation MRI (*t* = 1) or any subsequent follow-up (Fig. 1). For patients who received multiple treatments within one year, lesions were assessed when first detected. Monitoring continued for each lesion, even if the patient underwent an ablation unless the specific lesion itself was targeted. If this specific lesion was ablated, that lesion was no longer evaluated. Each lesion was individually assessed during follow-up evaluations. Each measurable tumor component at subsequent MRI was identified as a separate lesion, potentially resulting in more lesions than the original tumors. Imaging feature assessment was conducted using PACS (Sectra, Linköping) by a radiologist with 1 year of experience (DD), overseen by a radiologist with 15 years of experience (PG). Evaluated MRI features included [14, 16] (examples in Fig. 1):

- intermediate T2 signal [+iT2]*: hyperintense to muscle & hypointense to fluid; Present or Absent
- nodular morphology [+NOD]*: subjectively rounded appearance rather than the linear appearance of a lesion defined by a radiologist; Present or Absent
- contrast-enhancement [+ENH]*: subjective increase in signal at any time point on post-contrast SPGR, as compared to pre-contrast SPGR; Present or Absent
- maximal diameter (maxD)*: maximum diameter that was reproducibly measurable on all MRIs; measured in cm
- percent-monthly-change in diameter (PMCD)**: percent change in maxD divided by the time in months between *t* = 0 MRI and follow-up MRI being assessed; measured as % per month
  * =  applied to initial and subsequent MRIs
  **  =  applied only to *t* > 0 MRIs as *t* = 0 was the reference

**Fig. 2:**
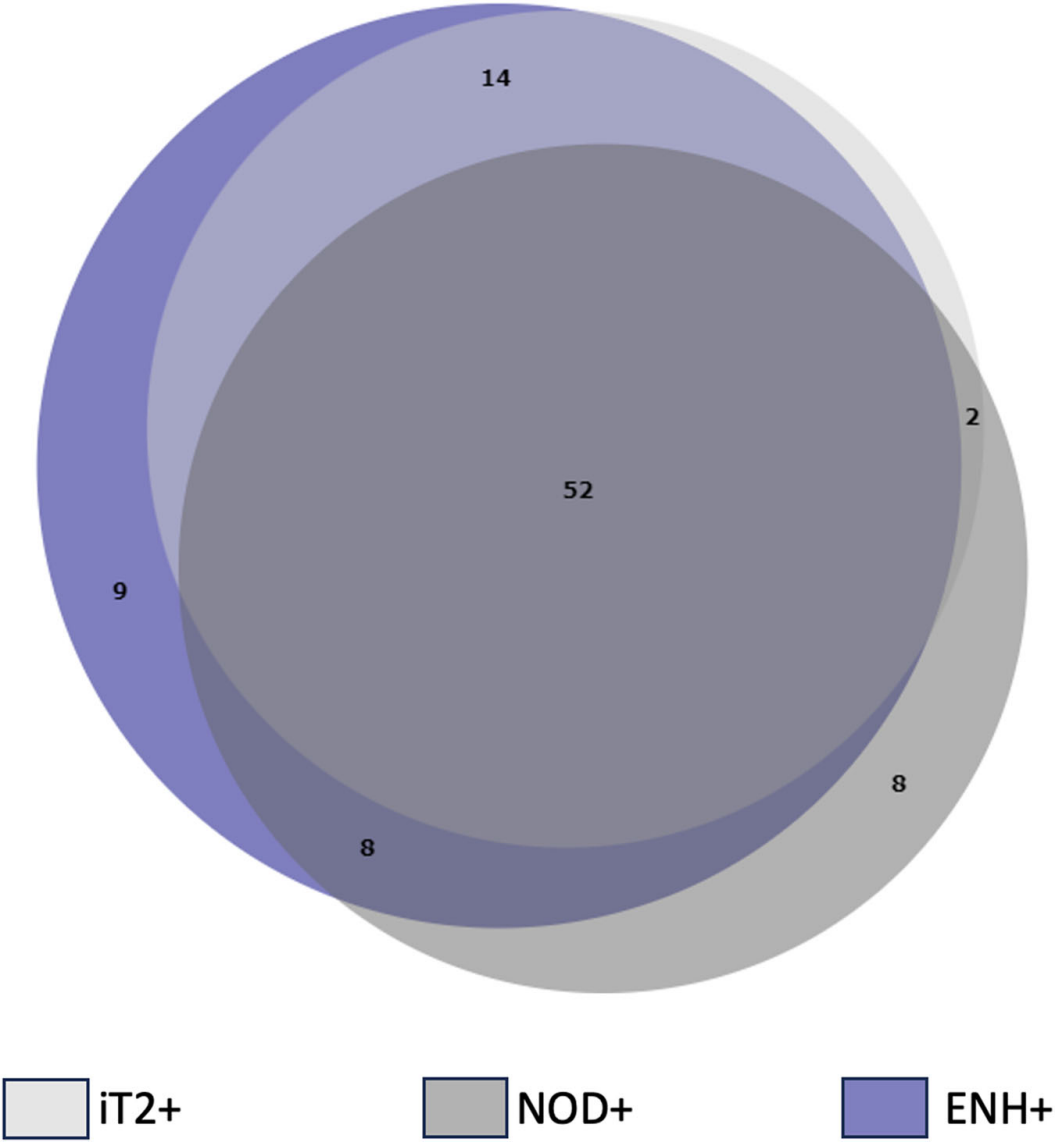
Venn diagram of groups of imaging features. This Venn diagram shows the groups +iT2 (*n* = 68), +ENH (*n* = 83) and +NOD (*n* = 70), and their subgroups, which were combinations of these three groups. The largest subgroup was +iT2 + NOD + ENH (*n* = 52), followed by +iT2 + ENH (*n* = 14). There were few examples of +iT2 + NOD (*n* = 2) and +NOD + ENH (*n* = 8), and no examples of −iT2

Patients were initially categorized based on single-variable features (Tables 1 and 2; Fig. 2), which were subsequently combined into six distinct categories (Table 3). Initial lesions may not have been assessed on immediate post-ablation imaging but could become visible and measurable in subsequent months. Lesions were graded into growing, unchanged, and regressing, based on criteria akin to those used by Gondim et al [14]: a positive PMCD of > 1% per month was defined as growing, a PMCD between −1 and +1% per month as unchanged, and a PMCD of < −1% per month as regressing. Non-progression was defined as cases that were unchanged or regressing. As this study did not assess treatment response, neither comparison to pre-treatment MRIs nor RECIST criteria were applicable.

**Table 1:** Correlation of MRI features with growth

A		
Overall lesion behavior	*n* (% total)	PMCD
Growing	62 (67%)	+5.6% IQR: 5.8
Unchanged	13 (14%)	−0.1% IQR: 0.6
Regressing	18 (19%)	−3.9% IQR: 4.2

B					
Lesions [+iT2]			Lesions [−iT2]		
**Totals**	**All cases**	***n*** **=** 68	**Totals**	**All cases**	***n*** = 25
	Growth	*n* = 59 (87%)		Growth	*n* = 3 (12%)
	Unchanged	*n* = 4 (6%)		Unchanged	*n* = 9 (36%)
	Regressing	*n* = 5 (7%)		Regressing	*n* = 13 (52%)
Lesions [+ENH]			Lesions [−ENH]		
**Totals**	**All cases**	***n*** = 83	**Totals**	**All cases**	***n*** = 10
	Growth	*n* = 62 (75%)		Growth	*n* = 0 (0%)
	Unchanged	*n* = 6 (7%)		Unchanged	*n* = 7 (70%)
	Regressing	*n* = 15 (18%)		Regressing	*n* = 3 (30%)
Lesions [+NOD]			Lesions [−NOD]		
**Totals**	**All cases**	***n*** = 70	**Totals**	**All cases**	***n*** **=** 23
	Growth	*n* = 52 (76%)		Growth	*n* = 10 (40%)
	Unchanged	*n* = 10 (15%)		Unchanged	*n* = 3 (12%)
	Regressing	*n* = 8 (12%)		Regressing	*n* = 10 (40%)

(A): Lesion grouped based on growth. A positive percent monthly change in diameter (PMCD) of > 1% was defined as growing, a PMCD between −1 and +1% as unchanged, and a PMCD of < −1% as regressing. The median PMCD of each category is summarized in the last column

(B): Lesions categorized based on each of the three imaging features iT2, ENH, and NOD, then grouped based on growth. Most of the [+iT2] or [+ENH] or [+NOD] lesions grew, while most of the [−iT2], [−ENH], or [−NOD] lesions did not grow

**Table 2:** Performance of individual MRI features in predicting growth vs. non-growth

	Sensitivity	Specificity	PPV	NPV	Accuracy
+iT2	95% (87–99%)	71% (52–86%)	87% (79–92%)	88% (70–96%)	87% (79–93%)
+Enh	100% (94–100%)	32% (17–51%)	75% (70–79%)	100% (69–100%)	77% (68–85%)
+Nod	84% (72–92%)	42% (25–61%)	74% (68–80%)	57% (39–72%)	70% (60–79%)

[+ENH] had the highest sensitivity and negative predictive value (NPV), while [+T2] had the highest specificity, positive predictive value (PPV), and accuracy for predicting growth over time. Data are presented as percentages with 95% confidence intervals in brackets

**Table 3:** Future tumor growth categorized by subgroup

Subgroup		PMCD (%)			ΔmaxD (cm)				Initial size (cm)		
Name	*n*	Median	Range	IQR	Median	Range	IQR	Time after *t* = 0	Median	Range	IQR
+iT2 + NOD + ENH	52	+5.9	−0.3; +29.6	6.2	+0.4	−0.1; +5.0	0.7	7.8 ± 3.7 months	1.9	0.4–16.5	1.9
+iT2 + ENH	14	+2.7	−2.9; +19.6	5.7	+0.1	−0.1; +0.5	0.2	9.0 ± 2.6 months	0.7	0.3–1.3	0.3
+iT2 + NOD	2	−2.6	−4.0; −1.3	1.3	−2.2	−4.4; −0.1	2.1	9.0 ± 4.2 months	4.7	2.6; 6.8	2.1
−iT2 + NOD + ENH	8	−2.1	−8.3; +4.0	4.5	−0.1	−0.5; +0.2	0.3	7.9 ± 3.9 months	1.5	0.3–3.2	1.4
−iT2 + ENH	9	−5.8	−33.3; +2.1	5.0	−0.2	−0.6; +0.1	0.3	7.0 ± 3.7 months	0.6	0.3–4.9	0.2
−iT2 + NOD	8	−0.4	−4.3; +0.2	0.4	−0.2	−0.9; ±0.0	0.4	9.4 ± 3.0 months	5.2	2.2–12.5	6.2

Patients were organized into subgroups based on different combinations of imaging features. The initial lesion size, percent monthly change in diameter (PMCD), and the average change in maximal diameter (Δ maxD) for each sub-group are illustrated here. The average change in maxD is assessed between the time = 0 MRI and the final follow-up MRI. There were no lesions that lacked both nodularity and enhancement

### Statistical analysis

GraphPad Prism (version 9 for macOS, GraphPad Software) was used to analyze all endpoints. Variables are expressed as the median, interquartile range (IQR), and range. Median PMCD values were compared between categories using Mann–Whitney or Kruskal–Wallis tests, followed by Dunn’s multiple comparison test. The aim was to identify MRI feature combinations correlating with positive PMCD of residual or recurrent DT components. Fisher’s exact test evaluated imaging features’ predictive performance for growth vs. non-growth. Differences were considered significant at *p* < 0.05.

## Results

The cohort included 33 patients (32 years ± 13.3; 22 females, 11 males) with a total of 34 DTs, who collectively underwent 26 MRgFUS and 21 cryoablation procedures. One patient had two desmoids treated with four HIFU treatments. Three patients were treated twice with HIFU, two patients underwent cryoablation (one with three and the other with four treatments), and two patients received both cryoablation and HIFU (one had one cryoablation and one HIFU, while the other had two cryoablations and one HIFU). Each patient underwent a median of 2 follow-up MRIs, with a median total follow-up duration of 269 days (range: 33–421 days) post-ablation. The median interval from treatment to the initial post-treatment MRI was 91 days (IQR: 19.5 days). A total of 93 discrete lesions were identified on post-ablation imaging and categorized as presented in Tables 1 and 2, and Fig. 2.

**Fig. 3:**
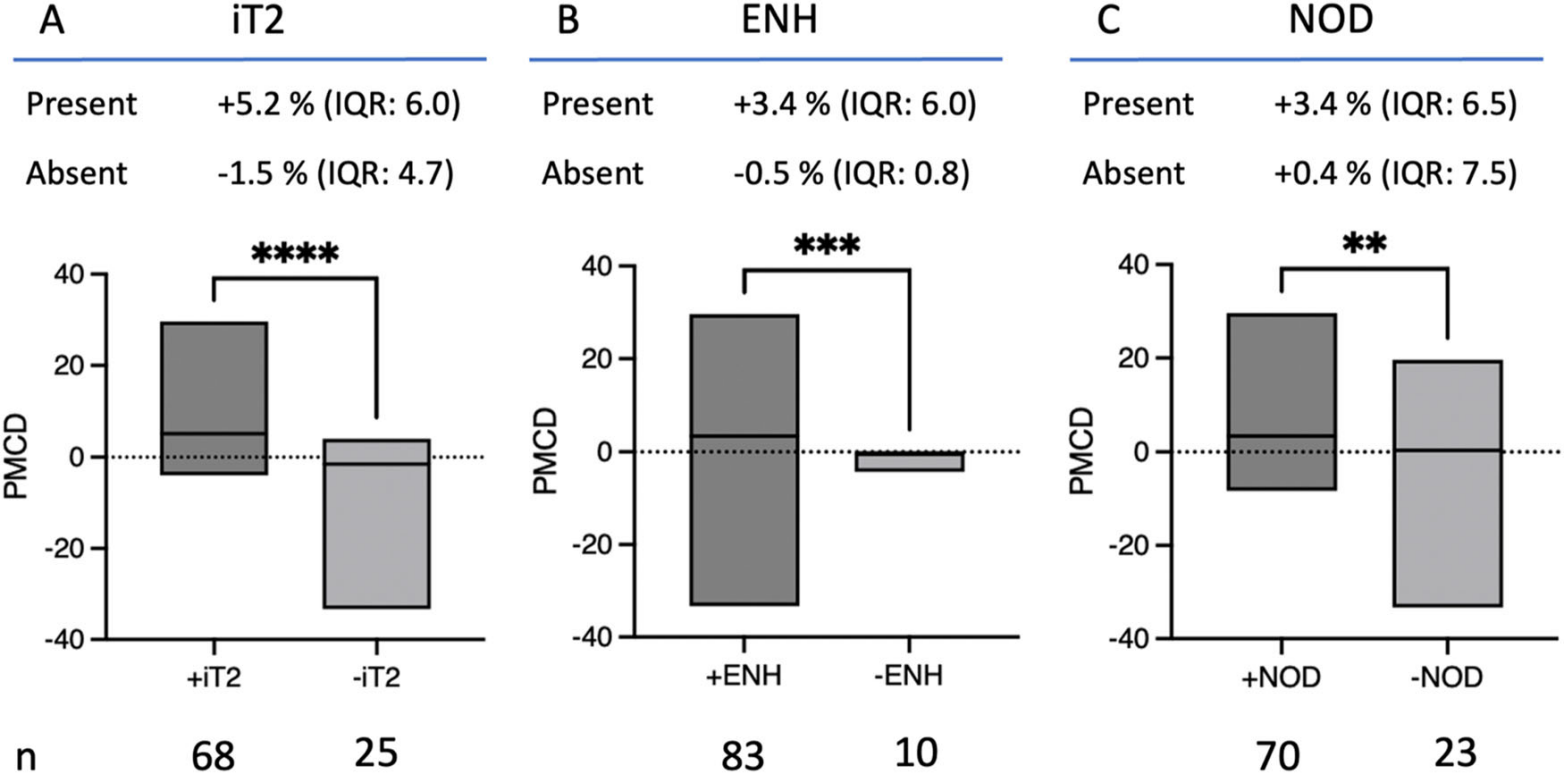
Differences in future tumor growth based on MRI features. Median percent monthly change in diameter (PMCD) is shown for lesions with (dark gray box) or without (light-gray box) each MRI imaging feature. **A** Intermediate T2 signal intensity [iT2], defined as hyperintense to background muscle and hypointense to fluid; **B** enhancement, defined as an increase in signal on post-contrast imaging relative to pre-contrast imaging [ENH]; **C** nodularity as assessed subjectively [NOD]. The box includes the range and median of PMCD. The number (*n*) and the median ± IQR of the percent monthly change in diameter (PMCD) of each group are stated

The PMCD of the groups defined by intermediate T2 signal, enhancement, and nodularity are illustrated in Fig. 3, alongside the distributions of growing, unchanged and regressing lesions within these groups (Table 1 and Fig. 3).

The [+iT2] group exhibited a positive PMCD in 91% (62/68), with 87% (59/68) of these lesions showing growth, whereas the [−iT2] group had a positive PMCD in 24% (6/25), with 12% (3/25) showing growth. The median PMCD was higher for [+iT2] lesions (+5.2% IQR: 6.0) compared to [−iT2] lesions (−1.5% IQR: 4.7; *p* < 0.0001). The presence of [+iT2] was a robust predictor of tumor growth (*p* < 0.0001), with a sensitivity of 0.95 (CI 95%: 0.87–0.99), specificity of 0.71 (CI 95%: 0.79–0.93), and accuracy of 0.87 (CI 95%: 0.79–0.93) (Table 2). Among the features assessed, [+iT2] demonstrated the highest specificity and accuracy for predicting tumor growth post-ablation.

Similarly, the [+ENH] group showed a positive PMCD in 81% (67/83), with 75% (62/83) of lesions demonstrating growth, while the [−ENH] group had a positive PMCD in 10% (1/10), with no lesions showing growth. The median PMCD was higher for [+ENH] lesions (+3.4% IQR: 6.0) compared to [−ENH] lesions (−0.5% IQR: 0.8; *p* = 0.003). The presence of [+ENH] predicted tumor growth (*p* < 0.0001), with a sensitivity of 1.00 (CI 95%: 0.94–1.00) and accuracy of 0.77 (CI 95%: 0.68–0.85), albeit with a lower specificity of 0.32 (CI 95%: 0.17–0.51) (Table 2).

For the [+NOD] group, a positive PMCD was observed in 79% (55/70), with 74% (52/70) of lesions showing growth, whereas the [−NOD] group had a positive PMCD in 57% (13/23), with 43% (10/23) showing growth. The median PMCD was higher for [+NOD] lesions ( + 3.4% IQR: 6.5) compared to [−NOD] lesions (+0.4% IQR: 7.5; *p* = 0.0056). The presence of [+NOD] predicted tumor growth (*p* = 0.01), with a sensitivity of 0.84 (CI 95%: 0.72–0.92) and accuracy of 0.70 (CI 95%: 0.60–0.79), but a specificity of 0.42 (CI 95%: 0.25–0.61) (Table 2).

**Fig. 4:**
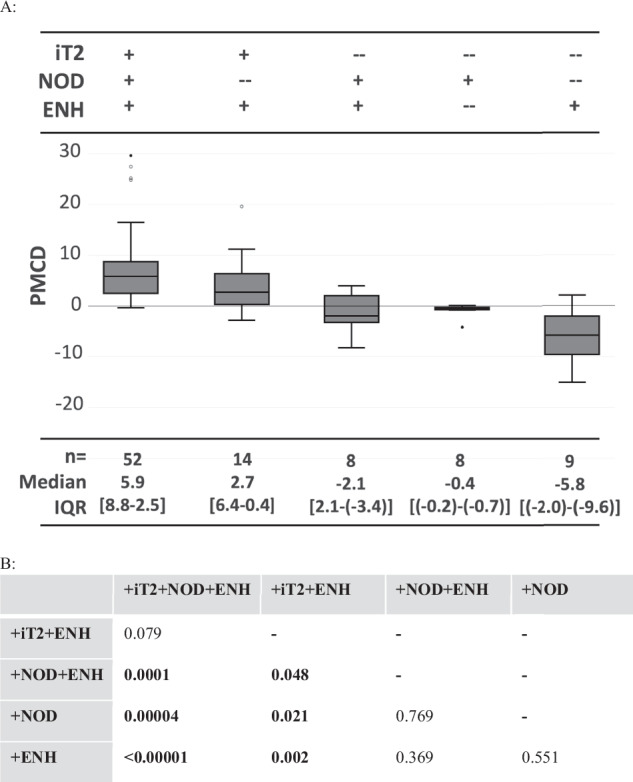
Comparison of subgroup performance. Kruskal–Wallis analysis showed that the subgroups are significantly different (*p* < 0.0001). **A** Box and whisker plots showing the percent monthly change in diameter (PMCD) of each subgroup; the median and interquartile range (IQR) are listed at the bottom. **B** Comparison of key subgroups showed that the PMCD for both [+iT2 + NOD + ENH] and [+iT2 + ENH] were significantly greater than all other subgroups, but were not significantly different between each other

**Fig. 5:**
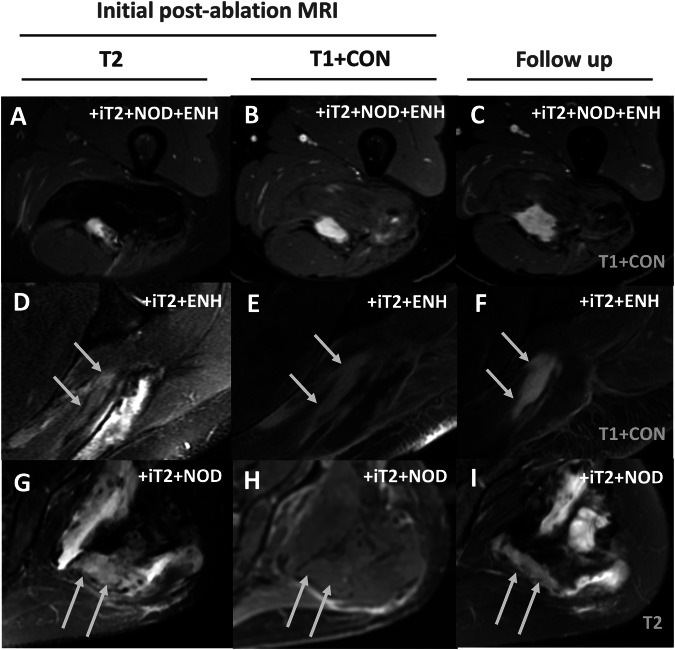
Examples of growing, unchanged, and regressing lesions in the context of the three subgroups with intermediate T2 signal. Column 1 is baseline T2 weighted FSE with fat saturation images (T2), while Column 2 is baseline T1 weighted SPGR fat-suppressed post-contrast images (T1 + CON). Column 3 is images from the last follow-up labeled as T2 or T1 + CON. (**A**–**C**: Top Row) 18-year-old female with a left posterior thigh desmoid tumor. Initial post-ablation imaging shows a nodule with intermediate T2 signal and enhancement. The nodule demonstrated growth on follow-up 365 days later. The patient underwent one HIFU and two cryoablations. (**D**–**F**: Middle Row) 59-year-old male with a left posterior shoulder desmoid tumor. Initial post-ablation MRI shows a non-nodular lesion with intermediate T2 signal and enhancement. The lesion was unchanged at follow-up 280 days following cryoablation. (**G**–**I**: Bottom Row) 55-year-old female with a left buttock desmoid tumor. Initial post-ablation MRI shows a non-enhancing nodule with an intermediate T2 signal. The nodule showed regression at follow-up 242 days later. The patient underwent two HIFU and one cryoablation. HIFU, high-intensity focused ultrasound under MRI guidance

Lesion categories were further combined into groups with various combinations of these three features (Tables 3 and 4; Figs. 4 and 5). Note that there were no lesions with [+iT2] that lacked both [ENH] and [NOD].

**Table 4:** Subgroup performance in predicting future growth vs. non-growth

Subgroup	Sens.	Spec.	PPV	NPV	Acc.
+iT2 + NOD + ENH	81% (69–90%)	94% (79–99%)	96% (87–99%)	71% (59–80%)	85% (76–92%)
+iT2 + ENH	15% (7–26%)	84% (66–95%)	64% (40–83%)	33% (29–37%)	38% (28–48%)
+iT2 + NOD^a^	0% (0–6%)	94% (79–99%)	0% (---)	32% (30–34%)	31% (22–42%)
−iT2 + NOD + ENH	3% (0–11%)	81% (63–93%)	25% (7–61%)	29% (26–44%)	29% (20–39%)
−iT2 + ENH	2% (0–9%)	74% (55–88%)	11% (2–49%)	27% (23–32%)	26% (17–36%)
−iT2 + NOD	0% (0–6%)	74% (55–88%)	0% (---)	27% (23–31%)	25% (16–35%)

This table shows percentages of sensitivity, specificity, positive and negative predictive value, and accuracy of the subgroups in differentiating lesions that will exhibit future growth vs. those that will not exhibit growth (either unchanged or regressing on future imaging). 95% confidence intervals are in brackets

^a^ This subgroup contains only two lesions, so the data are likely unreliable

The subgroup [+iT2 + NOD + ENH] exhibited the highest PMCD ( + 5.9% IQR: 6.2, *n* = 52), higher than all other combined subgroups (PMCD = −0.4% IQR: 2.6; *n* = 41; *p* < 0.0001; Fig. 4). Within the [+iT2 + NOD + ENH] subgroup, 98% (51/52) showed a positive PMCD, with 96% (50/52) meeting the growth criteria. Notably, one case of disease regression was concurrent with tamoxifen treatment for breast cancer. This subgroup demonstrated superior performance in distinguishing future growth from non-growth (Table 4 and Fig. 5) across all metrics, including sensitivity (0.81), specificity (0.94), positive predictive value (0.96), negative predictive value (0.71), and accuracy (0.85). The PMCD for the [+iT2 + NOD + ENH] subgroup was higher than all other subgroups (Fig. 5), except for [+iT2 + ENH] ( + 2.7% IQR: 5.7, *n* = 14, *p* = 0.079). Note that in some subgroups (e.g., +iT2 + ENH), the PMCD may be influenced by a smaller initial feature size (Table 3).

**Fig. 6:**
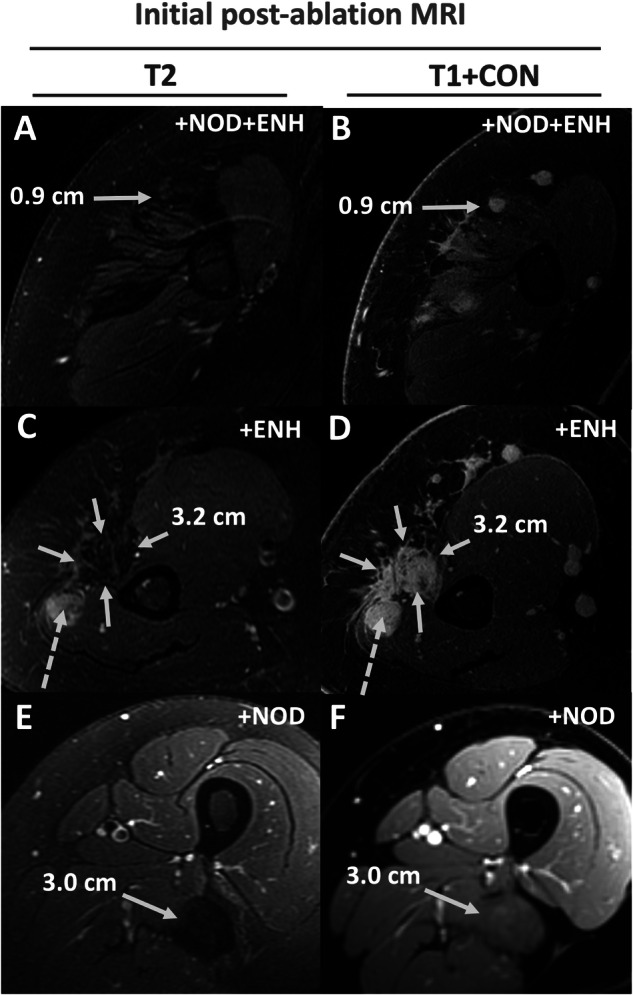
Examples of lesions from each subgroup without intermediate T2 signal. Column 1 is baseline T2 weighted FSE with fat saturation images (T2), while Column 2 is baseline T1-weighted SPGR fat- suppressed post-contrast images (T1 + CON). (**A**, **B**: Top Row) 22-year-old female with a proximal right arm desmoid tumor post cryoablation, showing an enhancing nodule without intermediate T2 signal. (**C**, **D**: Middle Row) 22-year-old female with a proximal right arm desmoid tumor post cryoablation, showing an area of non-nodular enhancement without intermediate T2 signal (solid arrows). Note there is an adjacent intermediate T2 enhancing nodule that was separately categorized (dashed arrow). (**E**, **F**: Bottom Row) Example from a treated left thigh desmoid tumor with a non-enhancing nodule that was T2 hypointense

PMCD was negative for the remaining subgroups (Table 3; Figs. 3 and 6), ranging from −0.37% IQR: 0.4 to –5.8% IQR: 5.0. The accuracy of subgroups other than [+iT2 + NOD + ENH] was low (range: 0.25–0.38), noting the smaller sample size in these groups (Table 3).

## Discussion

This study evaluated the predictive value of three MRI features (intermediate T2 signal, enhancement, and nodularity) in forecasting the growth of measurable lesions post-focal ablation of DTs. Intermediate T2 signal [+iT2] demonstrated high sensitivity (0.95) and accuracy (0.87) in predicting future tumor growth (PMCD: +5.2% IQR: 6.0), albeit with moderate specificity (0.71). The presence of either enhancement (PMCD: +3.4% IQR: 6.0) or nodularity (PMCD: +3.4% IQR: 6.5) is also strongly correlated with future growth. However, these features exhibited low sensitivity, positive predictive value (PPV), and negative predictive value (NPV) in the absence of iT2 signal, highlighting iT2 signal as the primary discriminator for growth post-ablation.

Areas of ill-defined enhancement post-ablation have been observed to be indicative of inflammation, e.g., after ablation of hepatocellular carcinoma [19]. In our experience, this is also frequently present after ablation of desmoids with both HIFU and cryoablation, which can persist for six months or longer, often appearing thick, nodular, or mass-like. This likely explains why enhancement and nodularity alone poorly predict future tumor growth, showing low specificities of 0.32 and 0.42, respectively. Nonetheless, when combined with iT2 signal, they contribute as supplementary factors influencing the balance between sensitivity and specificity. The presence of all three features predicted future lesion growth with excellent specificity (0.94), PPV (0.96), and highest accuracy (0.85), albeit at the expense of reduced sensitivity (0.81). This combination also exhibited the highest monthly growth rate (PMCD: +5.9% IQR: 6.2), surpassing other subgroups except for iT2 + enhancement.

A distinctive aspect of our findings compared to prior studies is the assignment of MRI features to discrete, measurable lesions within the treatment bed post-ablation, rather than assessing the entire tumor mass [13–17]. This distinction may reveal heterogeneous growth behaviors within tumors, influenced by varying cellular densities and perfusion levels [20, 21]. For example, often DTs display mainly low T2 signal, consistent with fibrosis, with a small proportion of the tumor appearing T2 hyperintense and enhancing [20, 21]; according to our data, this part of the tumor tends to grow and could be targeted for ablation.

Notably, prior research has yielded conflicting results regarding the correlation between T2 signal and future growth [13, 17, 18]. For instance, Libertini et al conducted subjective assessments of T2 signal changes following tamoxifen therapy in 32 DTs over a median follow-up of 45.5 months [13]. However, the methodology for their assessments was not detailed, leaving room for ambiguity regarding whether tamoxifen halts tumor growth without inducing fibrosis. Similarly, Kamali et al analyzed 90 extra-abdominal DTs, stratifying them by the extent of T2 hyperintensity, but found no correlation between T2 signal and growth over a median follow-up of 43 months, where half of the cases underwent surgical treatment [17]. Conversely, Cassidy et al reviewed 37 DTs managed with observation [15] and found that progression-free survival (PFS) at one year was 55% in tumors that were at least 90% T2 hyperintense vs. 94% in DTs with < 90% T2 hyperintensity. Gondim et al also reported a significant correlation between T2 signal intensity and growth in DTs with a T2 desmoid/muscle ratio > 1, demonstrating stabilization or regression in cases with a ratio < 1 [14]. Zhu et al studied 38 DTs treated with imatinib and observed a 55% PFS rate for lesions ≥ 75% T2 hyperintense vs. 83% for those < 75% T2 hyperintense [14, 16].

Limitations of our study include its retrospective design within a single center, primarily focusing on ablated DTs. Furthermore, the rarity of DTs resulted in small sample sizes for some MRI feature subgroups, particularly iT2 + nodularity, which included only two cases, one concurrently treated with tamoxifen. The predictive value of MRI features may diminish in patients receiving adjuvant therapies post-ablation. Future studies should incorporate dynamic contrast enhancement to distinguish between early and late enhancement, potentially improving specificity for early-enhancing lesions. Additionally, DTs exhibit varied prognoses depending on location, size, and patient age, which may influence findings within our cohort. Collectively, our findings support a short-term follow-up strategy based on PMCD thresholds, capturing even slow-growing tumors while informing decisions on patient monitoring and treatment escalation. Prospective studies with larger cohorts, extended follow-up durations, and comprehensive consideration of prior or concurrent therapies are warranted to refine follow-up strategies and enhance prognostic accuracy over longer timeframes.

In conclusion, our study suggests that defined MRI features within DT subcomponents offer insights into individual growth patterns and associated risks post-ablation. This information aids interventional radiologists and oncologists in evaluating treatment outcomes, monitoring patients, and planning subsequent interventions.

## Abbreviations

DT: Desmoid tumor
ENH: Enhancement
FOV: Field of view
HIFU: High-intensity focused ultrasound
IQR: Interquartile range
iT2: Intermediate T2 signal
maxD: Maximal diameter
mRECIST: Modified response evaluation criteria in solid tumors
MRgFUS: MR-guided high-intensity focused ultrasound
NOD: Nodularity
PFS: Progression-free survival
PMCD: Per monthly change
RECIST: Response evaluation criteria in solid tumors
TE: Echo time
TR: Repetition time
